# Association between *THRSP* Gene Polymorphism and Fatty Acid Composition in Milk of Dairy Cows

**DOI:** 10.3390/ani11041144

**Published:** 2021-04-16

**Authors:** Daniel Polasik, Jacek Golińczak, Witold Proskura, Arkadiusz Terman, Andrzej Dybus

**Affiliations:** 1 Department of Genetics, West Pomeranian University of Technology in Szczecin, al. Piastów 45, 70-311 Szczecin, Poland; arkadiusz.terman@zut.edu.pl (A.T.); andrzej.dybus@zut.edu.pl (A.D.); 2 Faculty of Biotechnology and Animal Husbandry, West Pomeranian University of Technology in Szczecin, Janickiego Street 32, 71-270 Szczecin, Poland; golinczakjacek@gmail.com (J.G.); witoldproskura@gmail.com (W.P.)

**Keywords:** dairy cattle, *THRSP*, polymorphism, milk, fatty acids

## Abstract

**Simple Summary:**

The purpose of this study was to detect polymorphism in thyroid hormone-inducible hepatic protein gene (*THRSP*) and analyze its influence on the fatty acid composition of milk in Jersey and Holstein-Friesian cattle. One single nucleotide polymorphism (SNP) was detected and determined in 224 cows. It was demonstrated that the analyzed variant had a significant influence on several fatty acids content in milk. Obtained results could be applied in breeding programs for improving the quality of milk.

**Abstract:**

Thyroid hormone-inducible hepatic protein is involved in the *de novo* synthesis of fatty acids in the lactating mammary gland. Different variants of the gene that encodes this protein may be associated with its different activity. The primary aim of this study was to find polymorphism in the *THRSP* gene and estimate the relationship between individual genotypes and fatty acid composition in milk. Investigations were carried out on 224 cows represented by two breeds—Jersey (*n* = 80) and Polish Holstein-Friesian (*n* = 144). Polymorphism in *THRSP* was detected by Sanger sequencing; however, genotypes were determined by the PCR-RFLP method. It was shown that the analyzed variant had a significant (*p* < 0.05) influence on palmitic and stearic fatty acids as well as on fatty acids with a chain length of 14, 16, and 6–16 in Jersey breed and on caproic, palmitic, myristoleic, and palmitoleic fatty acids in H-F. Obtained results indicated that analyzed SNP in bovine *THRSP* gene (rs42714482) may be considered as a potential marker for fatty acid composition in milk

## 1. Introduction

Milk fat is a source of fatty acids in the human diet, which can be beneficial for health or can be associated with the risk of some diseases [1]. The fatty acid profile can be modified by many factors; among them, environmental and genetic seems to be most important. Whereas environmental conditions can be managed by farmers if the genetic background is more complex to handle. First, we need to know which genes and their variants are correlated with fatty acid composition in milk. Therefore, it is important to analyze candidate genes for this trait, which can be typed based on the physiological role of the encoded protein, QTL mapping or GWAS.

Thyroid hormone-inducible hepatic protein gene (*THRSP*) encodes Spot14 (S14) protein, which is associated with regulation of the *de novo* fatty acid synthesis in the liver, adipose tissue, and lactating mammary gland [2]. It was shown that overexpression of *THRSP* in bovine mammary epithelial cells increased triacylglycerol levels and enhanced the expression of following lipogenic genes: fatty acid synthase (*FAS*), peroxisome proliferator-activated receptor γ (*PPARγ*) and sterol regulatory element-binding protein 1 (*SREBP1*) [3]. *THRSP* gene polymorphisms were investigated in many domestic animals. Analysis indicated that the *THRSP* gene in chicken is duplicated and 2 forms are present—*THRSPα* and *THRSPβ*. In both paralogs, insertion-deletion (indel) polymorphism was found. It was shown that *THRSPα* variants were correlated with the deposition of abdominal fat in chickens [4]. In another study, one SNP was detected in chicken *THRSPα* which together with earlier analyzed indel were associated with growth and body composition traits [5]. Similarly, detected SNP in the goat *THRSP* gene was studied in relation to growth traits. It was shown that different genotypes were correlated with body weight and chest girth in the Boer goat breed [6]. In pigs, however, SNP found in 5′ proximal regulating region of *THRSP* was associated with average backfat thickness, average daily weight gain, and loin-eye area [7]. Also in cows, polymorphism in the coding region of this gene was analyzed in relation to meat and carcass traits, as well as fatty acid composition in meat. It was shown that different *THRSP* genotypes were correlated with water holding capacity and meat tenderness in Qinchuan cattle and with unsaturated, monounsaturated, and few individual fatty acids in the meat of Hanwoo cattle [8,9]. In dairy cattle—Italian H-F, *THRSP* variants were associated with some milk production traits [10]. Bovine *THRSP* gene is located on chromosome 29 (Bta29) and consists of 2 exons. The length of the transcript is 1398 bp; however, the protein is composed of 148 aa [11,12]. Although the role of *THRSP* in the synthesis of fatty acids during lactation, polymorphism in the gene that encodes this protein was not studied in relation to the fatty acid composition of milk. Therefore this study aimed to detect polymorphism in the *THRSP* gene of Jersey and Polish Holstein-Friesian cattle and to perform association analysis for milk fatty acids profile.

## 2. Materials and Methods

### 2.1. Animals

The experiment covered 224 cows that belong to Jersey (*n* = 80) and Polish Holstein-Friesian (*n* = 144) breeds. The first group was reared in a tie-stall barn in Greater Poland Voivodeship; however, the second was reared in a free-stall barn in West Pomeranian Voivodeship. Feeding and management of animals on both farms were very similar. Cows were fed by use of a total mixed ration (TMR) diet that contains corn silage, grass haylage, alfalfa silage, straw, solvent-extracted soybean meal, as well as minerals and vitamins. The nutritional composition of feeds applied in the experiment is presented in Table 1. Blood samples were collected into tubes containing K_3_EDTA during a routine veterinary check-up. Milk samples were collected in lactations 1–4 during trial milking performed by the Polish Federation of Cattle Breeders and Dairy Farmers. The mean day of lactation for Holstein-Friesian cows was 195; however, for Jersey, it was 151.

### 2.2. Polymorphism Analysis

DNA was isolated from peripheral blood by use of MasterPure™ DNA Purification Kit for Blood Version II (Epicentre Biotechnologies, Madison, WI, USA). Following primers pair, covering bovine *THRSP* exon 1 was designed using Primer3 software [13]: F 5’-GCTGTGTTGACCTACTGGC-3’, R 5’-CGGCCACCATTACCTTTCCT3’. Primers were designed based on the ENSBTAG00000011666 sequence [10]. PCR cycling was as follows: initial denaturation at 94 °C/5 min, 35 cycles of 94 °C/30 s, 61 °C/45 s, 72 °C/30 s, and final extension at 72 °C/5 min. PCR was performed in a total volume of 15 µL that contains 50–80 ng of genomic DNA, 1.5 mM MgCl2, 0.3 mM of dNTP mix, 12 pmol of each primer, and 0.35 U of *Taq* polymerase (EURx, Gdansk, Poland). The presence of specific amplicons (598 bp) was confirmed in 1.5% agarose gel with Perfect™ 100–1000 bp DNA Ladder (EURx, Gdansk, Poland). Sequencing of amplicons was performed by an external service (Genomed, Warsaw, Poland). PCR-RFLP method was applied to determine detected *THRSP* gene variants (rs42714482) [14]. The same pair of primers and conditions as mentioned above were used in PCR. A total of 10 µL of obtained amplicons were digested by *BstC8*I enzyme (SibEnzyme, Novosibirsk, Russia) in 55 °C at least 3 h. Restriction fragments were separation in 4% agarose gels with a 50 bp DNA Ladder (Genoplast, Rokocin, Poland).

### 2.3. Milk Composition Analysis

To avoid a period of negative energy balance, milk samples were collected after the 90th day of lactation during morning milking, according to PN-EN ISO 5555:2002 standard. Next samples were transported to the laboratory and stored at −20 °C until further analysis. Lipids were extracted from milk by use of chloroform and methanol mixture in a 2:1 ratio. Next fatty acids were converted into methyl esters using boron trifluoride according to PN-EN ISO 12966-2:2011 standard. Analysis of fatty acids methyl esters was performed using gas chromatography mass-spectroscopy method (GC-MC) in agreement with PN-EN ISO 5508:1996 standard. Fatty acids were identified based on their relative retention time in relation to retention times of standard (SupelcoTM 37 Component FAME Mix, Sigma-Aldrich, Saint Louis, MI, USA). Following fatty acids were analyzed:

- saturated: C6:0 (caproic), C8:0 (caprylic), C10:0 (capric), C12:0 (lauric), C14:0 (myristic), C16:0 (palmitic), C18:0 (stearic);
- unsaturated: C14:1 (myristoleic), C16:1 (palmitoleic), C18:1n-9c (oleic), C18:1n-9t (elaidic), C18:2n-6c (linoleic), C18:3n3 (α-linoleic).

Peaks were analyzed by use of TurboMassTM software (PerkinElmer, Waltham, MA, USA).

Additionally, the following indexes were calculated: sum of fatty acids with a chain length of 14 (ΣC14), 16 (ΣC16), 6–16 (ΣC6–16), 18 (ΣC18), Δ9-desaturase index for fatty acid with 14 carbons (Δ9IC14), 16 (Δ9IC16), 18 (Δ9IC18), for monounsaturated fatty acids (Δ9MUFA), saturated fatty acids (SFA), unsaturated fatty acids (UFA), monounsaturated fatty acids (MUFA), polyunsaturated fatty acids (PUFA), UFA/SFA ratio, atherogenic index (AI) and thrombogenic index (TI). The Δ9 desaturase and atherogenic/thrombogenic indices were calculated using the formulas proposed by Lock and Garnsworthy [15] and Ulbricht and Southgate [16].

### 2.4. Statistical Analysis

Statistical analysis was conducted using R packages [17]. Pedigree data were arranged in Pedigree Viewer, ver. 6.5 (University of New England, Armidale, Australia) [18]. An additive relationship matrix based on a three-generation pedigree was generated using the kinship2 R package (Rochester, MI, USA) [19]. To estimate associations between individual *THRSP* genotypes and analyzed traits following mixed linear model was estimated and applied using the lmekin function from the coxme R package (Rochester, MI, USA) [20]:

Y = µ + G + LS + β1A + β2DLC + α + e

where: Y—the value of the analyzed trait, µ—overall mean, G—fixed effect of *THRSP* genotype, LS—fixed effect of lactation number and season, β1A—regression coefficient for the age of cow, β2DLC—regression coefficient for the day of lactation when milk was collected, α—random polygenic effect taking into consideration pedigree relationships, e—the random error. Statistical significance of results was indicated below a *p*-value of 0.05 (*p* < 0.05).

## 3. Results

**Figure 1:**
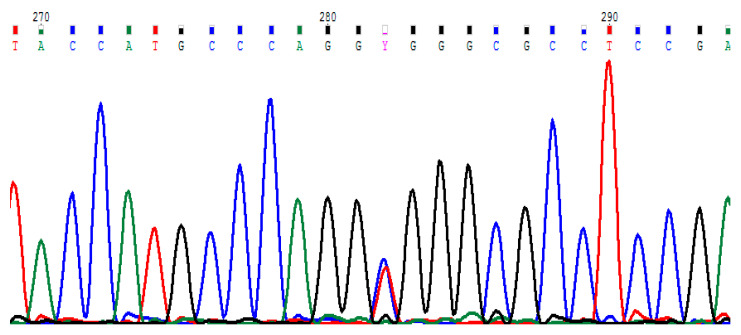
Sequencing results showing the polymorphic site in the bovine *THRSP* gene, Y—heterozygous genotype (*CT*).

Sequencing of bovine *THRSP* exon 1 allowed detection of one missense SNP located in position 193 of the transcript (152 in cds) (Figure 1). This C/T substitution led to alanine to valine exchange in position 51 of the protein.

**Figure 2:**
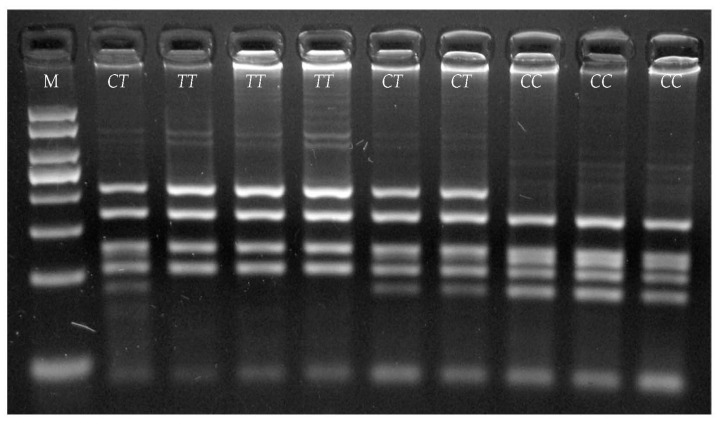
Determination of *THRSP* genotypes by use of PCR-RFLP method, M—50 bp DNA Ladder (50, 100, 150, 200, 250, 300, 400, 500 bp) (Genoplast, Poland).

Restriction analysis showed that the BstC8I enzyme can differentiate detected variants in the *THRSP* gene. Individual genotypes were determined based on the following restriction fragments lengths: *TT*—205, 164, 124, 105 bp, *CT*—205, 164, 124, 116, 105, 89 bp, *CC*—164, 124, 116, 105, 89 bp (Figure 2).

The distribution of *THRSP* genotypes and alleles with Hardy-Weinberg equilibrium (HWE) in both analyzed breeds of cows are presented in Table 2. It was shown that TT genotype was most frequent in Jersey (0.33) group; however, CC was most frequent in Polish H-F (0.46). In the case of alleles, reverse tendency was observed where T was the major allele in Jersey cows (0.58), while C in H-F (0.68). Analysis showed that the distribution of genotypes was in agreement with the HWE expectation. Frequencies of individual genotypes were significantly different between Jersey and Polish Holstein-Friesian breeds (χ^2^ = 29.442, *p* < 0.01)

Fatty acid composition of milk with some indexes in relation to *THRSP* variants is shown in Table 3 and Table 4. In Jersey cows, statistically significant differences (*p* < 0.05) were found between individual genotypes and following milk fatty acids: palmitic, stearic as well as following indexes: fatty acids with a chain length of 14, 16, and 6–16. In Polish H-F cows, associations (*p* < 0.05) were found for caproic, palmitic, myristoleic, and palmitoleic fatty acids in milk. In both breeds, only one trait was common as correlated with *THRSP* genotypes—palmitic fatty acid. Jersey cows with *TT* genotypes were characterized by the highest value; however, Polish H-F with the same variant had the lowest.

## 4. Discussion

*THRSP* is a nuclear protein that can regulate lipogenesis [21]. It was found that *THRSP* may regulate milk fat synthesis by directly affecting the activity of some classical lipogenic enzymes [3]. Recently, Salcedo-Tacuma et al. [22] indicated *THRSP* as an inhibitor of lipid synthesis in adipose tissue of periparturient Holstein cows.

*THRSP* gene is involved in *de novo* fatty acid synthesis. The fatty acids that are synthesized *de novo* belong to short-chain and medium-chain length acids, from C4 to C14 and also some C16. The C18 fatty acids and some C16, however, arise from the plasma lipids [23]. In our study, we analyzed fatty acids from C6 to C18. We decided to include C18 fatty acids because an earlier report showed a significant decrease in C18:0 upon *THRSP* overexpression in goat mammary epithelial cells [24].

*THRSP* gene in Polish H-F and Jersey breeds in relation to milk fatty acids composition was analyzed for the first time. In a previous study, Oh et al. [9] selected 8 SNPs in the bovine *THRSP* gene from GenBank [13] for analysis in Korean cattle (Hanwoo). Only two of them, namely g.78 G > A and g.184 C > T were polymorphic in this breed. Comparison of sequences from Ensembl [11] and GenBank [14] showed that the g.184 C > T variant is the same as detected in our work (29:17994763 C/T, rs42714482). Analyzed polymorphism causes amino acid substitution in position 51 of the encoded transcription factor, which probably may modify its interaction with regulated genes and, thus, differentiate processes connected with fat milk synthesis, including the composition of individual fatty acids in milk. We found that the frequency of individual genotypes in Korean cattle (*CC*—0.17, *CT*—0.46, *TT*—0.37) was very similar to those observed in our study in Jersey breed. Evolutionary analysis of SNPs located in genes of Hanwoo cattle in relation to data from other cattle breeds, i.e., Jersey, Simmental, Angus, and Holstein showed that the Korean breed was distinctly separated from the other four breeds. Further SNPs analysis, however, showed that the *THRSP* gene is not classified as Hanwoo-specific, which may reflect similar genetic parameters in Korean and Jersey breeds [25]. When comparing the frequency of *THRSP* genotypes in Italian Holstein-Friesian cattle (*CC*—0.48, *CT*—0.42, *TT*—0.10), the same was observed in our work for Polish Holstein-Friesian breed [10].

As mentioned earlier, the *THRSP* gene was not investigated in relation to milk fatty acids. It was studied, however in relation to carcass traits and fatty acid composition of muscle fat in Korean Cattle, as well as to health and milk production traits in Italian Holstein cattle. It was shown that g.184 C > T SNP is significantly correlated with myristic, palmitic, myristoleic, oleic, linoleic, linolenic fatty acids in *m. longissimus dorsi*, as well as with saturated fatty acids, monounsaturated fatty acids, and monounsaturated/saturated ratio [9]. In our study, we found an association between *THRSP* genotypes and palmitic fatty acid in milk of Jersey and Polish H-F cows and myristoleic fatty acid in HF cows. In the muscle of Hanwoo and the milk of Jersey cattle, the highest content of palmitic fatty acid was found for the *TT* genotype. In the Polish H-F breed, however, a reverse tendency was observed, because the *TT* genotype was correlated with its lowest value. Similarly, *TT* genotype was favorable for myristoleic fatty acid in the muscle of Hanwoo cows but in the milk of Polish Holstein-Friesian cows, it was disadvantageous. These differences may result from fatty acids content in milk of particular cattle breeds. A significant breed effect on the content, fatty acid profile and atherogenic or thrombogenic properties of milk fat was reported by Sobotka et al. [26]. The concentrations of long-chain saturated fatty acids were significantly higher in the milk fat of Jersey cows than in the H-F breed. The milk of H-F cows had lower fat content but provided more health benefits than the milk of Jersey. It was also confirmed by the analysis of Jersey, H-F, and three other breeds reared in the Netherlands. Breed differences were found for individual fatty acids, among them for palmitic and myristoleic [27]. We cannot also exclude that observed differences in association tendency may be linked with different rearing conditions for both breeds. We found also an association of *TT* genotypes with higher content of caproic and myristoleic fatty acid in Polish H-F and with lower stearic fatty acid in Jersey, but this relationship was not observed in Hanwoo cattle. Analysis of *THRSP* gene in Italian Holstein cows also showed a significant association for milk yield, milk fat yield, milk protein yield and productivity, functionality, and type index, with allele *T* being favorable for these traits [10]. In our study, we did not find any relationships between individual *THRSP* genotypes and milk yield, fat yield, and fat content both in Polish Holstein-Friesian and Jersey breeds. Similarly, *THRSP* genotypes were not found to influence the atherogenic and thrombogenic indexes. As mentioned earlier, we also noticed significant associations for stearic, caproic, palmitoleic fatty acids and fatty acids with a chain length of 14, 16, and 6–16. Stearic acid is common in nature, both in animal and vegetable organisms; however, its level is usually higher in animal than vegetable fat [28]. In milk, it arises from the plasma lipids, but interestingly, the highest expression of *THRSP* is correlated with its decrease in goat mammary epithelium [24]. In the Jersey breed, *TT* genotype was associated with the lowest level of stearic acid in milk. Caproic, caprylic, and capric fatty acids are known as the reason for the specific aroma of goat and sheep milk [29]. Analysis of caproic acid amount in the milk of different species showed its highest level in goat milk, lowest in sheep; however, in cow milk, it was slightly higher than in sheep [30]. In Polish Holstein-Friesian cows, the *TT* variant of *THRSP* related to the highest content of this fatty acid. Palmitoleic acid shows anti-inflammatory and antidiabetic activity and is produced by desaturation of palmitic acid [31]. In Polish H-F animals we found that *TT* genotype is correlated with the lowest amount of palmitoleic, as well as earlier mentioned palmitic fatty acid, which can reflect their metabolic relationships. In the case of confirmed associations of *THRSP* polymorphism and fatty acids with a chain length of 14, 16, and 6–16 in Jersey cattle, we obtained unclear results. The highest value of the first trait was found for the *CC* genotype; however, the second and third traits were found for the *TT* genotype.

## 5. Conclusions

The conducted experiment showed that *THRSP* polymorphism (rs42714482) is associated with milk fatty acid composition in Jersey and Polish Holstein-Friesian cattle. It covers palmitic, stearic caproic, myristoleic, and palmitoleic fatty acids, as well as fatty acids with a chain length of 14, 16, and 6–16 in a particular breed. Only palmitic acid content was the common trait for both breeds, however, with opposite tendency, which may reflect breed differences or/and feeding and housing conditions. Analyzed SNP in bovine *THRSP* gene could be taken into consideration as a potential marker for fatty acid composition in milk.

## Figures and Tables

**Table 1:** Nutritional composition of feeds.

Parameter		Breed	
		Holstein-Friesian	Jersey
Dry weight	%	45.0	40.7
Protein	g/kg dw	149.9	157.4
Fat	g/kg dw	32.6	29.8
Carbohydrates	g/kg dw	763.3	727.7
Calcium	mg/kg dw	6.0	9.0
Magnesium	mg/kg dw	2.9	3.4
Phosphorus	mg/kg dw	2.8	4.7
Natrium	mg/kg dw	2.6	2.7

**Table 2:** Distribution of *THRSP* genotypes and alleles in analyzed breeds of cows.

Breed	*n*	Genotypes			Alleles		HWE	
		*CC*	*CT*	*TT*	*C*	*T*	χ^2^	*p*
Jersey	80	0.16 (*n* = 13)	0.51 (*n* = 41)	0.33 (*n* = 26)	0.42	0.58	0.223	0.637
Polish Holstein-Friesian	144	0.46 (*n* = 66)	0.45 (*n* = 65)	0.09 (*n* = 13)	0.68	0.32	0.282	0.596

HWE—Hardy-Weinberg equilibrium.

**Table 3:** Association of *THRSP* genotypes with analyzed traits of milk in Jersey cows.

Trait	Genotype			Mean	*p*
	*CC* (*n* = 13)	*CT* (*n* = 41)	*TT* (*n* = 26)		
MY	22.646 ± 4.653	20.917 ± 3.749	22.323 ± 4.142	21.655 ± 4.053	n.s.
FY	1.193 ± 0.232	1.049 ± 0.191	1.114 ± 0.245	1.093 ± 0.220	n.s.
FC	5.306 ± 0.544	5.045 ± 0.624	4.995 ± 0.702	5.072 ± 0.640	n.s.
C6:0	2.913 ± 0.433	2.626 ± 0.377	2.694 ± 0.370	2.695 ± 0.392	n.s.
C8:0	1.877 ± 0.331	1.637 ± 0.238	1.653 ± 0.296	1.681 ± 0.284	n.s.
C10:0	3.553 ± 0.292	3.407 ± 0.431	3.425 ± 0.453	3.437 ± 0.418	n.s.
C12:0	4.173 ± 0.389	3.928 ± 0.539	3.901 ± 0.609	3.959 ± 0.545	n.s.
C14:0	12.716 ± 0.873	12.351 ± 1.211	12.241 ± 1.320	12.375 ± 1.198	n.s.
C16:0	37.197 ± 2.523 ^a^	37.346 ± 3.207	38.462 ± 3.028 ^b^	37.684 ± 3.062	*p* < 0.05
C18:0	12.516 ± 1.461	12.883 ± 1.599 ^b^	12.242 ± 1.726 ^a^	12.615 ± 1.627	*p* < 0.05
C14:1	1.517 ± 0.396	1.299 ± 0.351	1.323 ± 0.423	1.342 ± 0.386	n.s.
C16:1	1.588 ± 0.235	1.543 ± 0.282	1.710 ± 0.444	1.605 ± 0.342	n.s.
C18:1n-9c	15.703 ± 1.959	17.001 ± 3.496	16.569 ± 3.716	16.650 ± 3.370	n.s.
C18:1n-9t	1.092 ± 0.168	1.021 ± 0.204	1.039 ± 0.220	1.038 ± 0.203	n.s.
C18:2n-6c	2.119 ± 0.321	2.018 ± 0.334	1.900 ± 0.389	1.996 ± 0.355	n.s.
C18:3n-3	0.114 ± 0.042	0.118 ± 0.050	0.103 ± 0.045	0.113 ± 0.047	n.s.
ΣC14	14.233 ± 1.093 ^b^	13.650 ± 1.439	13.564 ± 1.583 ^a^	13.717 ± 1.441	*p* < 0.05
ΣC16	38.786 ± 2.466 ^a^	38.889 ± 3.152	40.172 ± 3.057 ^b^	39.289 ± 3.048	*p* < 0.05
ΣC6–16	62.622 ± 3.452	61.511 ± 4.809 ^a^	62.717 ± 4.770 ^b^	62.083 ± 4.590	*p* < 0.05
ΣC18	31.557 ± 3.396	33.055 ± 5.002	31.865 ± 5.106	32.424 ± 4.807	n.s.
Δ9IC14	9.458 ± 3.342	9.065 ± 2.775	10.052 ± 3.308	9.450 ± 3.042	n.s.
Δ9IC16	5.734 ± 2.214	4.847 ± 1.417	4.839 ± 1.569	4.988 ± 1.628	n.s.
Δ9IC18	65.067 ± 7.090	63.796 ± 5.739	65.410 ± 3.995	64.527 ± 5.472	n.s.
Δ9MUFA	23.154 ± 2.379	24.049 ± 3.908	23.703 ± 4.181	23.791 ± 3.772	n.s.
SFA	77.304 ± 2.314	76.467 ± 3.776	76.839 ± 4.176	76.724 ± 3.694	n.s.
UFA	22.696 ± 2.314	23.533 ± 3.776	23.161 ± 4.176	23.276 ± 3.694	n.s.
MUFA	20.160 ± 2.120	21.135 ± 3.560	20.913 ± 3.928	20.904 ± 3.479	n.s.
PUFA	2.536 ± 0.394	2.398 ± 0.416	2.248 ± 0.438	2.372 ± 0.426	n.s.
UFA/SFA	0.295 ± 0.039	0.311 ± 0.069	0.305 ± 0.077	0.307 ± 0.067	n.s.
AI	4.125 ± 0.661	3.986 ± 0.878	4.095 ± 0.907	4.044 ± 0.849	n.s.
TI	5.250 ± 0.739	5.183 ± 0.987	5.324 ± 0.978	5.240 ± 0.940	n.s.

MY—milk yield [kg], FY—fat yield [kg], FC—fat content [%], SFA—saturated fatty acids, UFA—unsaturated fatty acids, MUFA—monounsaturated fatty acids, PUFA—polyunsaturated fatty acids, AI—atherogenic index, TI—thrombogenic index; ^a,b^—means with different superscripts in the same row differ statistically significant at *p* < 0.05, n.s.—not significant.

**Table 4:** Association of *THRSP* genotypes with analyzed traits of milk in Polish Holstein-Friesian cows.

Trait	Genotype			Mean	*p*
	*CC* (*n* = 66)	*CT* (*n* = 65)	*TT* (*n* = 13)		
MY	31.344 ± 8.441	30.434 ± 8.711	30.877 ± 7.975	30.891 ± 8.478	n.s.
FY	1.267 ± 0.369	1.266 ± 0.350	1.264 ± 0.383	1.266 ± 0.359	n.s.
FC	4.067 ± 0.598	4.221 ± 0.663	4.098 ± 0.678	4.140 ± 0.635	n.s.
C6:0	2.296 ± 0.442	2.173 ± 0.420 ^a^	2.458 ± 0.470 ^b^	2.255 ± 0.440	*p* < 0.05
C8:0	1.333 ± 0.241	1.278 ± 0.247	1.372 ± 0.288	1.312 ± 0.248	n.s.
C10:0	3.097 ± 0.510	2.988 ± 0.581	3.169 ± 0.730	3.054 ± 0.564	n.s.
C12:0	3.712 ± 0.554	3.656 ± 0.708	3.756 ± 0.774	3.691 ± 0.644	n.s.
C14:0	12.478 ± 1.342	12.170 ± 1.592	12.379 ± 1.381	12.330 ± 1.461	n.s.
C16:0	41.211 ± 5.073	41.450 ± 4.590 ^b^	39.644 ± 5.248 ^a^	41.177 ± 4.867	*p* < 0.05
C18:0	9.377 ± 2.416	8.871 ± 2.541	10.269 ± 2.699	9.229 ± 2.514	n.s.
C14:1	1.321 ± 0.462	1.443 ± 0.521 ^b^	1.107 ± 0.372 ^a^	1.357 ± 0.490	*p* < 0.05
C16:1	2.062 ± 0.633	2.352 ± 0.758 ^b^	1.849 ± 0.606 ^a^	2.174 ± 0.707	*p* < 0.05
C18:1n-9c	16.275 ± 2.863	16.660 ± 3.609	17.012 ± 3.400	16.515 ± 3.253	n.s.
C18:1n-9t	0.962 ± 0.291	1.046 ± 0.327	0.896 ± 0.165	0.994 ± 0.302	n.s.
C18:2n-6c	2.865 ± 0.679	2.718 ± 0.618	3.180 ± 0.737	2.827 ± 0.666	n.s.
C18:3n-3	0.280 ± 0.099	0.280 ± 0.087	0.310 ± 0.106	0.283 ± 0.094	n.s.
ΣC14	13.799 ± 1.490	13.613 ± 1.798	13.486 ± 1.342	13.687 ± 1.619	n.s.
ΣC16	43.274 ± 5.066	43.801 ± 4.705	41.493 ± 5.249	43.351 ± 4.929	n.s.
ΣC6–16	67.511 ± 4.836	67.509 ± 5.456	65.733 ± 5.704	67.350 ± 5.190	n.s.
ΣC18	29.772 ± 4.810	29.588 ± 5.701	31.680 ± 5.718	29.861 ± 5.305	n.s.
Δ9IC14	9.515 ± 2.952	10.493 ± 3.352	8.263 ± 2.846	9.844 ± 3.184	n.s.
Δ9IC16	4.824 ± 1.563	5.393 ± 1.647	4.511 ± 1.519	5.053 ± 1.619	n.s.
Δ9IC18	63.698 ± 5.355	65.449 ± 5.205	62.483 ± 6.037	64.379 ± 5.411	n.s.
Δ9MUFA	23.766 ± 3.896	24.625 ± 3.980	24.245 ± 4.534	24.197 ± 3.986	n.s.
SFA	75.709 ± 3.746	74.937 ± 3.897	75.112 ± 4.524	75.306 ± 3.877	n.s.
UFA	24.291 ± 3.746	25.063 ± 3.897	24.888 ± 4.524	24.694 ± 3.877	n.s.
MUFA	20.901 ± 3.286	21.805 ± 3.702	21.135 ± 4.013	21.330 ± 3.547	n.s.
PUFA	3.391 ± 0.782	3.259 ± 0.715	3.753 ± 0.874	3.364 ± 0.768	n.s.
UFA/SFA	0.324 ± 0.069	0.338 ± 0.074	0.336 ± 0.082	0.332 ± 0.072	n.s.
AI	4.017 ± 0.785	3.860 ± 0.800	3.902 ± 1.052	3.936 ± 0.815	n.s.
TI	4.922 ± 0.953	4.725 ± 0.893	4.777 ± 1.185	4.820 ± 0.947	n.s.

MY—milk yield [kg], FY—fat yield [kg], FC—fat content [%], SFA—saturated fatty acids, UFA—unsaturated fatty acids, MUFA—monounsaturated fatty acids, PUFA—polyunsaturated fatty acids, AI—atherogenic index, TI—thrombogenic index; ^a,b^—means with different superscripts in the same row differ statistically significant at *p* < 0.05, n.s.—not significant.

## Data Availability

Data is contained within the article.
